# Laboratory Evolution of Fast-Folding Green Fluorescent Protein Using Secretory Pathway Quality Control

**DOI:** 10.1371/journal.pone.0002351

**Published:** 2008-06-11

**Authors:** Adam C. Fisher, Matthew P. DeLisa

**Affiliations:** 1 School of Chemical and Biomolecular Engineering, Cornell University, Ithaca, New York, United States of America; 2 Department of Biomedical Engineering, Cornell University, Ithaca, New York, United States of America; Université de Montréal, Canada

## Abstract

Green fluorescent protein (GFP) has undergone a long history of optimization to become one of the most popular proteins in all of cell biology. It is thermally and chemically robust and produces a pronounced fluorescent phenotype when expressed in cells of all types. Recently, a superfolder GFP was engineered with increased resistance to denaturation and improved folding kinetics. Here we report that unlike other well-folded variants of GFP (e.g., GFPmut2), superfolder GFP was spared from elimination when targeted for secretion via the SecYEG translocase. This prompted us to hypothesize that the folding quality control inherent to this secretory pathway could be used as a platform for engineering similar ‘superfolded’ proteins. To test this, we targeted a combinatorial library of GFPmut2 variants to the SecYEG translocase and isolated several superfolded variants that accumulated in the cytoplasm due to their enhanced folding properties. Each of these GFP variants exhibited much faster folding kinetics than the parental GFPmut2 protein and one of these, designated superfast GFP, folded at a rate that even exceeded superfolder GFP. Remarkably, these GFP variants exhibited little to no loss in specific fluorescence activity relative to GFPmut2, suggesting that the process of superfolding can be accomplished without altering the proteins' normal function. Overall, we demonstrate that laboratory evolution combined with secretory pathway quality control enables sampling of largely unexplored amino-acid sequences for the discovery of artificial, high-performance proteins with properties that are unparalleled in their naturally occurring analogues.

## Introduction

The green fluorescent protein (GFP) from *Aequorea* jellyfish [1] is inefficiently folded when expressed in *Escherichia coli* [2], [3]. To address this problem, several folding-enhanced variants of GFP have been created over the years that have effectively elevated GFP to one of the most studied and exploited proteins in biochemistry and cell biology [2], [4]. Recently, two studies reported the creation of GFP superproteins. Superproteins are best defined as high-performance proteins that ignore at least some biologically imposed restrictions on amino acid sequence and occupy regions of sequence space unexplored by naturally occurring proteins optimized for *in vivo* function [5], [6]. For instance, Waldo and coworkers reported the engineering of a superfolder GFP (sfGFP) that showed improved tolerance to circular permutation, increased resistance to denaturation, improved folding kinetics, and increased resistance to aggregation during refolding [7], [8]. sfGFP has proven to be very useful as a scaffold for improved protein detection and tagging both *in vivo* and *in vitro* using self-assembled sfGFP fragments [9], [10] and for peptide insertions that confer binding activity to sfGFP giving rise to so-called fluorobodies [11]. Along similar lines, Liu and coworkers recently engineered supercharged versions of sfGFP whose net charge was altered by as much as +48 charge units and, as a result, remained soluble even when exposed to conditions that strongly favored aggregation [12].

Remarkably, almost ten years elapsed between the publication of the sfGFP sequence and the original folding-optimized ‘cycle 3’ mutant GFP [2]. This notable lag led us and others [13] to question whether this was due to inherent difficulties in engineering folding-enhanced variants of GFP or whether methods of screening and selecting were limiting progress. Indeed, the creation of both sfGFP and supercharged GFP revealed the potential for engineering protein folding properties that surpassed those of their parental sequences. For example, a simple folding interference screen was used to evolve sfGFP [7] from the ‘folding reporter’ variant of GFP (frGFP) [14], that contained the cycle 3 mutations [2] and the ‘enhanced GFP’ mutations F64L and S65T [15]. Importantly, several folding properties including improved folding kinetics and resistance to aggregation were achieved without disruption of the protein's normal function. In fact, expression of sfGFP was accompanied by a two-fold increase in cellular fluorescence [7]. Likewise, in the case of the supercharged GFP variants, resistance to aggregation was improved significantly without affecting the proteins' ability to fold or fluoresce [12]. In general, by surpassing the physical and chemical properties of naturally occurring proteins, such superproteins offer several advantages including, for instance, increased capabilities as biosensors [16] and FRET partners [9]; improved fidelity as transcription reporters [17]; and improved therapeutic properties (e.g., longer systemic half-life, improved efficacy) [6].

To expand the superprotein engineering toolbox, here we sought to develop a general strategy for the discovery of superfolded proteins. Specifically, we explored the hypothesis that native cellular quality control pathways exert sufficient evolutionary pressure to improve protein folding properties beyond those that are normally sufficient for solubility and function within a cell. Along these lines, we previously developed a genetic selection for protein solubility based on the inherent folding quality control mechanism of the twin-arginine translocation (Tat) pathway that results in exclusive export of correctly folded, soluble proteins across the inner membrane in *E. coli* [18], [19]. A similar and often overlooked protein folding quality control also exists for the general secretory (Sec) pathway (for a review see [20]). The Sec export pathway is the most utilized secretion pathway in nearly all bacteria [21]. Substrates of this pathway pass through the SecYEG translocase and are exported in a post-translational manner with the chaperone SecB, the signal peptide, and translocase itself maintaining substrates in largely unfolded conformations to minimize premature protein folding prior to translocation [22], 23, 24. Substrate proteins that fold prematurely in the cytoplasm and become resistant to unfolding are often refractory to post-translational Sec transport and are either degraded or, if sufficiently stable, accumulate in the cytoplasm [25], [26], [27], [28], [29]. An ostensibly co-translational mode of SecYEG-mediated transport is also possible via nascent substrate interaction with the signal recognition particle (SRP) [30]. Here we report that the protein folding quality control of SecYEG-mediated secretion is capable of effectively discriminating between well-folded and superfolded versions of GFP and, as a result, can be used for the engineering of superfolded GFP variants with properties that are unparalleled in their naturally occurring counterparts.

## Results

### Exploring the use of cellular folding quality control to identify superfolded proteins

**Figure 1:**
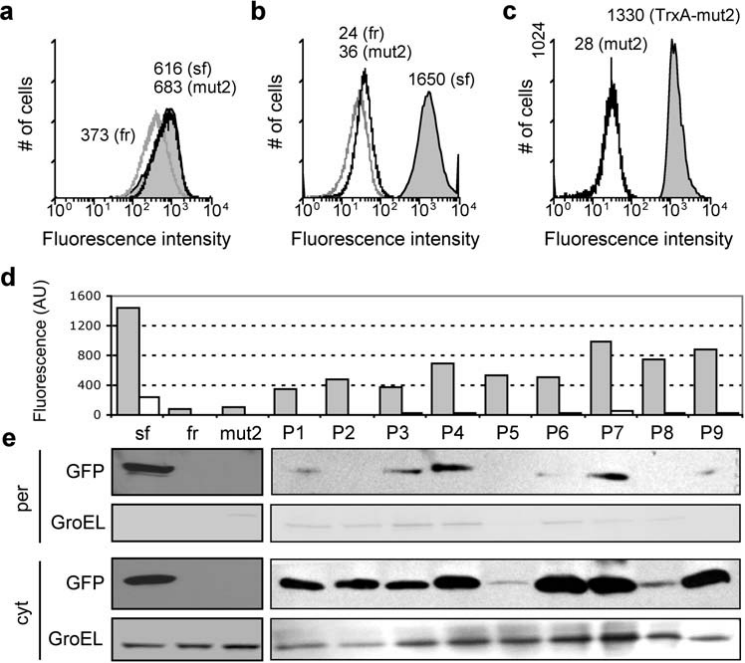
Flow cytometric analysis of GFP variants. Fluorescence histograms for cells expressing: superfolder GFP (sf, gray fill), folding reporter GFP (fr, gray line), and GFPmut2 (mut2, black line) (a) without a signal peptide and a C-terminal 6xhis tag or (b) as an N-terminal fusion to the ssDsbA signal peptide; (c) an N-terminal ssDsbA signal peptide fused to GFPmut2 (black line) and TrxA-GFPmut2 (gray fill). The geometric mean is listed next to each histogram. There was no significant difference in growth rate between any of the cultures (data not shown). (d) Fluorescence (arbitrary units) and subcellular localization of GFP as measured for cytoplasmic (cyt, grey bars) and periplasmic (per, white bars) fractions generated from cells expressing the various ssDsbA-GFP fusions including sf, fr, mut2 and clones P1–P9. (e) Western blot analysis of the per and cyt fractions of cells expressing the same fusions probed with GFP antiserum. Blots were probed with anti-GroEL serum as a fractionation marker.

To facilitate directed evolution of superfolded proteins, our initial goal was to develop a cellular screen for easily differentiating between cells expressing frGFP and sfGFP. To begin, we examined cytoplasmic expression of frGFP and sfGFP. Despite the fact that the folding properties of sfGFP are far superior to those of frGFP, there was only a small increase (65%) in the geometric mean fluorescence of cells expressing sfGFP relative to frGFP (Fig. 1a). This increase was due mostly to an increase in the specific fluorescence of sfGFP rather than an increase in soluble expression for the better folding mutant (see Table 1). Since we desired a more pronounced difference in fluorescence emission between sfGFP and frGFP to facilitate directed evolution by fluorescence activated cell sorting (FACS), we explored whether protein quality control associated with either the Tat or SecYEG-mediated export pathways might be capable of yielding a more striking difference in fluorescence for sfGFP and frGFP. While the Tat pathway is known to modulate export efficiency of a substrate based on folding and solubility [18], [19], owing to the similar *in vivo* solubility profiles of sfGFP and frGFP we observed only a modest phenotypic difference between cells expressing sfGFP and frGFP when each was targeted to the Tat pathway (data not shown). Alternatively, we reasoned that proteins targeted for SecYEG export would experience one or more of the following fates: (a) accumulation in the periplasm if transport via SecYEG was successful; (b) degradation in the cytoplasm if transport failed and the protein was sensitive to proteolysis due to insufficient folding or stability; and (c) accumulation in the cytoplasm if transport failed but the protein was resistant to unfolding and proteolysis. Additionally, for GFP export, the latter scenario is the only one that would be expected to give rise to cellular fluorescence because GFP that is routed into the periplasm through SecYEG is non-fluorescent [31].

**Table 1:** Yield, activity and folding parameters for the various GFPs.

GFP variant	Culture yield (mg/L)	Fluorescence yield (AU)	Equilibrium unfolding, C_1/2_ (M)	Kinetic unfolding, t_1/2_ (min)	Kinetic refolding, t_1/2_ (min)
frGFP	13.8+/−0.4	5.55+/−0.01	4.37+/−0.02	1.7+/−0.7	73+/−5
sfGFP	13.4+/−0.2	7.10+/−0.04	4.84+/−0.04	1.5+/−0.2	20+/−2
GFPmut2	16.3+/−0.4	5.06+/−0.04	3.40+/−0.06	0.4+/−0.3	33+/−2
P4	15.0+/−0.4	4.42+/−0.02	3.76+/−0.03	0.5+/−0.2	17+/−3
P5	15.5+/−0.3	4.38+/−0.01	3.79+/−0.04	6+/−1	22+/−1
P7 (superfast)	15.0+/−0.1	3.61+/−0.04	5.06+/−0.04	20+/−3	11+/−1

To experimentally test this notion, we targeted sfGFP and frGFP for translocation via SecYEG by N-terminal fusions of each to the DsbA (ssDsbA), maltose binding protein (ssMBP), and alkaline phosphatase (ssPhoA) signal peptides. These three signal peptides are derived from native *E. coli* substrates whose export is SRP-dependent [30], SecB-dependent [32], and SecB-independent [33], respectively. Upon induced expression of each fusion, we observed a 67-, 47- and 24-fold greater fluorescence emission for cells expressing ssDsbA-sfGFP, ssMBP-sfGFP and ssPhoA-sfGFP fusions, respectively, relative to the corresponding frGFP fusions (shown in Fig. 1b for ssDsbA and Supplemental Fig. S1a and b for ssMBP and ssPhoA). We suspected that the strong cellular fluorescence for sfGFP was a result of cytoplasmic accumulation for this extremely well-folded protein. Indeed, subcellular fractionation revealed that the fluorescence emitted by cells expressing the sfGFP fusions was localized predominantly in the cytoplasmic fraction (Fig. 1d) and that all of the sfGFP constructs accumulated at a high level in the cytoplasm (Fig. 1d and e, Supplemental Fig. S1c). It should be noted that a measurable amount of both ssDsbA-sfGFP and ssMBP-sfGFP localized in the periplasm but as expected [31], this material was largely inactive. Though we were initially surprised that sfGFP was able to accumulate in the cytoplasm when targeted for co-translational export via the SRP-dependent ssDsbA, we suspect that this was due to saturation of the SRP machinery [34] and not because of re-routing to a post-translational (e.g., SecB) export pathway (see also Supplemental Fig. S1d) as seen earlier for SRP routing of MBP [30]. Because cytoplasmic ssDsbA-sfGFP folds very rapidly and is resistant to unfolding, it is likely that cellular quality control factors (e.g., proteases) are unable to eliminate this protein and it accumulates in a fluorescent conformation. On the other hand, ssDsbA-frGFP is efficiently degraded and does not accumulate anywhere in cells (Supplemental Fig. S1e). Taken together, the differential cytoplasmic stability of SecYEG-targeted sfGFP and frGFP suggests that quality control can be used to easily discriminate the folding behavior of these variants.

### Exploiting SecYEG-mediated quality control to engineer folding-enhanced proteins

To determine whether SecYEG-mediated quality control exerts sufficient evolutionary pressure for engineering superproteins, we attempted to evolve a superfolded version of the FACS-optimized GFP variant known as GFPmut2. To create GFPmut2, Cormack *et. al.* introduced random mutations to only 20 amino acids flanking the chromophore of wildtype GFP and successfully isolated a well-folded, FACS-optimized GFP variant (S65A/V68L/S72A) [4]. Indeed, the fluorescence of cells expressing GFPmut2 in the cytoplasm was nearly identical to that of cells expressing sfGFP (Fig. 1a). However, cells expressing GFPmut2 targeted to SecYEG via ssDsbA were 46-fold less fluorescent than cells expressing ssDsbA-sfGFP (Fig. 1b) and were comparable to the low fluorescence seen earlier for those expressing ssDsbA-frGFP. Notably, cells expressing ssMBP-GFPmut2 and ssPhoA-GFPmut2 were also 36-fold and 4-fold less fluorescent, respectively, than the corresponding sfGFP (Supplementary Figure S1a, b).

**Figure 2:**
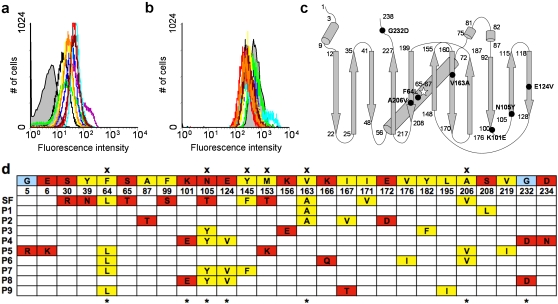
Phenotype and genotype characterization of isolated GFPmut2 variants. Fluorescence histograms for cells expressing (a) N-terminal ssDsbA fusions and (b) C-terminal 6xhis tag fusions to GFPmut2 (gray fill) and the variants P1 (black); P2 (green), P3 (blue), P4 (purple), P5 (light blue), P6 (yellow), P7 (red), P8 (brown) and P9 (orange). There was no significant difference in whole-cell fluorescence for cells expressing the various GFPs with or without the 6xhis tag (data not shown). (c) Schematic representation of GFP scaffold with the 7 recurring substitutions highlighted with black circles. In addition, all variants carry the GFPmut2 substitutions (S65A/V68L/S72A). (d) Substitutions found in clones P1–P9 with the 7 recurring substitutions represented by crosses (top) and the shared superfolder mutations represented by asterisks (bottom).

Next, we sought to enhance the folding of ssDsbA-GFPmut2 so that it would accumulate in the cytoplasm. Our first approach was via direct fusion of GFPmut2 to thioredoxin-1 (TrxA), an extremely well-folded protein known to fold too rapidly for Sec transport [30] and known to enhance fusion protein solubility [35]. As expected, cell fluorescence was restored following expression of a tripartite fusion of ssDsbA-TrxA-GFPmut2 (Fig. 1c); supporting our hypothesis that folding-enhancement of SecYEG-targeted GFPmut2 can mediate cytoplasmic accumulation of that protein. Encouraged by these observations, we next attempted to evolve a folding-enhanced version of GFPmut2 by rescuing cytoplasmic accumulation of SecYEG-targeted variants. This approach accentuated the dependence of the fluorescent phenotype on *in vivo* folding and was in stark contrast to the more common strategy of evolving fluorescent proteins by optimizing *in vivo* fluorescence from the direct expression of GFP, such as was initially done for GFPmut2 [4] and ‘cycle 3’ GFP [2]. Because ssDsbA fusions resulted in the greatest difference in fluorescence between cells expressing sfGFP and GFPmut2 (46-fold), we chose ssDsbA for our library evolution experiments. We cloned a high-rate error-prone DNA library (4.8% amino acid error) of GFPmut2 variants downstream of ssDsbA resulting in a diverse cell library of ∼1.5×10^6^ variants. Following two rounds of FACS we isolated 9 colonies that displayed fluorescent phenotypes significantly above the continuum distribution. The sequences of these variants revealed that: (a) all were full-length GFPmut2 variants fused in-frame to ssDsbA; and (b) all were unique with distinct patterns in the amino acid substitutions (Fig. 2d).

Of the 35 total substitutions that were found, 7 specific substitutions occurred more than once and accounted for 19 of the 35 total substitutions. These recurring substitutions with frequencies in parenthesis were: N105Y (4×), F64L (4×), E124V (3×), K101E (2×), A206V (2×), G232D (2×) and V163A (2×). Some of the substitutions even appeared in combination multiple times, for example N105Y/E124V (3×) and even K101E/N105Y/E124V/G232D (2×). In just these 9 clones, we isolated mutations that were uncovered in each previous folding-enhanced variant of GFP: V163A is a cycle 3 mutation (we also found M153K) [2], F64L is an enhanced GFP mutation [15], A206V and Y145F are superfolder mutations [7] and our most common mutation, N105Y, is found at the same residue as the N105T superfolder mutation. Of the 11 reported substitutions in sfGFP, we isolated mutations at 6 identical residues suggesting that our collection of GFPmut2 variants represented *bona fide* folding-enhanced versions of GFPmut2.

### Characterization of the folding-enhanced GFPmut2 variants

To test whether these variants were in fact enhanced for folding, we first re-transformed the recovered plasmids (clones P1–P9) into fresh cells and observed that re-transformed cells expressing variants P1–P9 all showed increased fluorescence relative to ssDsbA-GFPmut2 (Fig. 2a) as a result of cytoplasmic accumulation of each variant (Fig. 1d, e). We subcloned the variants appended with a C-terminal 6x-histidine tag but without the coding region for ssDsbA and observed that cells expressing these signal sequence-less GFPmut2 variants showed little to no loss in fluorescence activity when compared to the parental GFPmut2 protein (Fig. 2b), indicating that any improvement in GFP folding (see below) was accomplished without significantly affecting the proteins' normal function.

**Figure 3:**
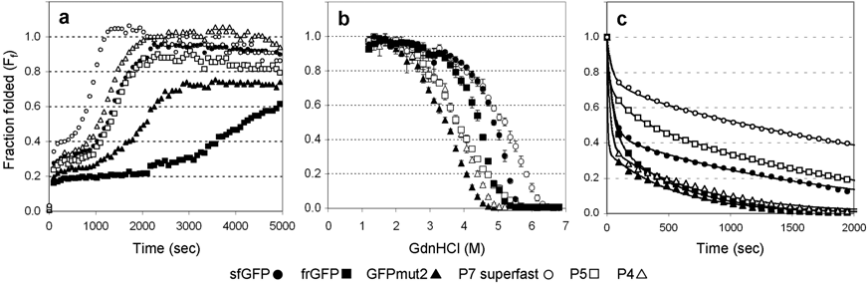
*In vitro* characterization of GFP folding. (a) Kinetic refolding measured as fraction folded (F_f_) over time, (b) equilibrium unfolding measured as F_f_ versus GdnHCl molarity and (c) kinetic unfolding measured as F_f_ over time for the different GFP variants as indicated. Curve fits were added as a visual aid. All data is the average of three replicate experiments where the standard error for all data was <10%.

We next chose to characterize clones P4, P5 and P7 in greater detail because cells expressing ssDsbA fused to each yielded the three highest whole-cell fluorescence values and collectively contained six of the seven recurring substitutions mentioned above. Following purification, we observed that P4, P5, and P7 had a slightly lower soluble yield and slightly lower total fluorescence relative to GFPmut2 (Table 1), suggesting that these clones represent a novel solution to protein folding optimization as neither the function nor the soluble yield of the protein-of-interest was improved. Since it has been shown in the past that fast-folding proteins can be trapped in the cytoplasm during Sec transport [30] and since sfGFP folds faster than frGFP [7], we reasoned that a logical explanation for the cytoplasmic accumulation of P4, P5, and P7 was increased folding kinetics. Indeed, following complete unfolding, the refolding speed of variants P4 and P5 was comparable to, while P7 far eclipsed, that of sfGFP (Fig. 3a, Table 1). Since sfGFP was one of the fastest folding GFPs to date [7], we renamed P7 ‘superfast’ GFP. In addition, P4, P5, superfast GFP, and sfGFP all approached complete recovery during refolding while frGFP and GFPmut2 stalled around 60% and 75% recovery, respectively.

Further, since mutations at N105 and A206 were previously shown to have no effect on folding kinetics, we wondered if these variants displayed increased resistance to chemical denaturation [7]. We examined equilibrium unfolding and discovered that P4, P5 and superfast GFP displayed increased stability relative to GFPmut2 in the presence of GdnHCl (Fig. 3b, Table 1). Surprisingly, frGFP displayed a modest stability during equilibrium unfolding suggesting that the evolved variants were not isolated based on surpassing a threshold of equilibrium unfolding stability. Intrigued by this observation, we examined the kinetics of unfolding and discovered that superfast GFP and P5 unfolded very slowly whereas P4 unfolded at a speed comparable to GFPmut2 (Fig. 3c, Table 1). Thus, while we conclude that each of these variants appears to have been isolated primarily based on improved folding kinetics, laboratory evolution returned three unique responses to the pressure imposed by secretory quality control: improved folding kinetics (P4); improved folding kinetics and slower unfolding (P5); and improved folding kinetics, slower unfolding, and increased stability during equilibrium unfolding (superfast GFP).

## Discussion

Numerous lines of evidence have revealed the existence of a multi-component folding quality control system that regulates the export of proteins out of the bacterial cytoplasm [20]. One instance of this sort of quality control exists for Tat export, which we previously exploited for the development of a high-throughput genetic selection for protein solubility [19]. In the present study, we have demonstrated that laboratory evolution combined with the folding quality control inherent to SecYEG-mediated export enables sampling of largely unexplored amino-acid sequences for the discovery of artificial, high-performance proteins with properties that are unparalleled in their naturally occurring analogues. As briefly demonstrated with TrxA (see Fig. 1c), we are optimistic that the SecYEG-mediated folding quality control may extend to at least some other protein targets. In this case, a general strategy for evolving folding-enhanced versions of these proteins may be possible by assaying for cytoplasmic accumulation of a target protein fused to a reliable cytoplasmic reporter protein [36], [37], protein fragment [9], [38], [39], or peptide [40], [41] that could be coupled with SecYEG targeting to create superfolders on demand.

Despite the potential for isolating superfolder GFPs, an unresolved question that arises is: what are the underlying cellular factors that govern whether a substrate is degraded or retained in the cytoplasm, especially for non-native substrates? Historically, it has been observed that during secretion various heterologous proteins are rapidly degraded [25] and various proteases play a role in this process [42], [43]. Recently, de Gier and coworkers observed that overexpressed native SRP substrates accumulate in the cytoplasm by titrating out targeting components (*e.g.* SRP and FtsY); these ‘overflow’ substrates become susceptible to degradation and inclusion body formation in the cytoplasm [34]. We observed a similar overflow in that the fluorescence of cells expressing ssDsbA fused to P4, P5, and P7 was dependent on overexpression, as constitutive expression from the *trc* promoter (i.e., no inducer) showed no appreciable cell fluorescence (data not shown). It is possible that certain overflow SRP substrates, particularly membrane proteins, accumulate in the cytoplasm by forming inclusion bodies. Overflow ssDsbA-sfGFP may accumulate in the cytoplasm by folding rapidly to evade degradation, while overflow ssDsbA-frGFP is efficiently eliminated. Thus, there likely exists a kinetic competition between folding and degradation of overflow SRP substrates, suggesting that the cytoplasmic accumulation of an SRP-dependent protein is linked to both SRP saturation and quality control. As such, care needs to be taken to avoid further saturating the quality control machinery. The ability to broadly exploit this quality control feature in the future will likely depend on optimizing promoter/induction conditions. There are several aspects of SecYEG-mediated secretion that may play a concerted role in the quality control of GFP (e.g. interactions with the signal peptide or translocon, chaperone or protease recruitment, compatibility with the inner membrane). However one thing is certain, when fast-folding GFP mutants are appended with a signal peptide targeting them for secretion via SecYEG, these proteins accumulate in the cytoplasm and confer a strong fluorescent phenotype to cells that is not present in the case of non-superfolding GFPs.

Finally to answer our initial query: is it inherently difficult to engineer folding-enhanced variants of GFP or are methods of screening and selecting lacking? Based on the relative ease with which we engineered folding-enhanced variants of GFPmut2, the answer appears to be a lack of robust screening methods. For instance, a single round of mutagenesis and two rounds of FACS yielded superfolded clones P4, P5, and superfast GFP that each exhibited significant folding-enhancement relative to the parental GFPmut2 protein. However, the isolation of these clones would not have been possible without the exploitation of SecYEG-mediated quality control as a screening platform; rather, we would have remained stalled at the self-imposed ‘glass-ceiling’ of functional advantage.

## Materials and Methods

### Bacterial strains and plasmids

*E. coli* strain MC4100 was used for all experiments, except for the library which was in DH5α. All plasmids were derivatives of pTrc99A (Amp^R^) (Amersham Pharmacia). For protein expression, GFP variants were appended with a 6xHis tag and cloned between *Xba*I and *Hin*dIII. The coding sequences for the signal peptides were PCR amplified from MC4100 chromosomal DNA and cloned into pTMB (Cm^R^) [19] between *Sac*I and *Xba*I. The GFP variants were cloned between *Xba*I and *Hin*dIII. The coding regions for GFPmut2 [4], frGFP [14], and sfGFP [7] were PCR amplified from pTGS [44], pCS-GFP (kindly provided by G. Waldo) [14], and pCR4Blunt-TOPO-superfolder_GFP (Geneart AG), respectively. Libraries of GFPmut2 were synthesized using the mutagenic method of Fromant *et al*. [45] and inserted in-frame with the coding region for the DsbA signal peptide. Plasmids were confirmed by DNA sequencing. Antibiotic selection was maintained at: ampicillin, 100 µg/ml; chloramphenicol (Cm), 20 µg/ml.

### Cell culture and *in vivo* fluorescence analysis

Overnight cultures were diluted 1∶100 into fresh LB medium with Cm or Amp at 37°C and induced with 100 µM IPTG in the early-exponential phase. At 4 h of induction, 10 µL of cells were diluted into 1 ml of PBS and analyzed by a flow cytometer (FACSCalibur; Becton Dickinson Biosciences). All flow cytometric analysis was performed with 488 nm excitation. For fluorescence activated cell sorting (FACS), the population was gated by side scatter (488/10 nm), forward scatter (488/10 nm), and fluorescence emission (530/30 nm) windows. Approximately ∼4.1×10^6^ DH5α library cells (diversity ∼1.5×10^6^) were subjected to FACS. The collected solution was sterile filtered (0.45-µm pore; Millipore), and placed on LB/Cm plates. 3,023 colonies were recovered, scraped, and inoculated into a fresh culture for a second round of FACS. 192 individual colonies were recovered in 200 µL LB/Cm in 96-well plates. The next day, cells were diluted 1∶100 into LB/Cm/IPTG, grown, and screened via a fluorescent microplate reader (Bio-Tek Synergy HT; Bio-Tek Instruments); excitation 485/20 nm, emission 528/20 nm. Plasmids were recovered and sequenced from selected clones.

### Protein isolation

Cultures were normalized by absorbance (600 nm) and fractionated using the ice-cold osmotic shock procedure [18], [46]. Western blotting of these fractions was performed [18]. Samples were read on a fluorescent microplate reader. For *in vitro* folding, native protein was purified on Ni-NTA columns according to the manufacturer's specifications (Ni-NTA Fast Start Kit, QIAGEN). Samples were concentrated and recovered in PBS buffer according to manufacturer's specifications (VivaSpin 6, Viva Science). SDS-PAGE was performed to verify binding efficiency. A Bio-Rad Protein Assay was used to quantify purified protein (Bio-Rad).

### Protein folding analysis

Equilibrium unfolding experiments were performed in 96-well plates at a protein concentration of 33 µg/mL with guanidinium chloride (GdnHCl) in TNG buffer (25 mM Tris [pH 7.5], 0.2 M NaCl, 5% glycerol) supplemented with 1mM dithiothreitol (DTT). C_1/2_ values were determined by fitting the curves at 24 h in Microsoft Excel as described [7]. Three trials were averaged and the error bars represent plus or minus the standard deviation. Manual mixing kinetic unfolding experiments were performed by rapid dilution into 6.8 M GdnHCl TNG buffer (33 µg/mL protein). Manual mixing kinetic refolding experiments were performed by rapid 10-fold dilution of fully unfolded samples into fresh TNG buffer (1 mM DTT) without GdnHCl (3.3 µg/mL protein and 0.68 M GdnHCl). The initial readings were taken prior to addition of buffer and every eleven seconds thereafter (dead time = 21 s). Three trials were averaged. For readability, every seventh data point is shown and the standard error is not shown (avg. <10%). t_1/2_ values were determined by fitting the unfolding kinetic curves to double exponential decay (frGFP, sfGFP, GFPmut2, P4) and triple exponential decay (P5 and superfast GFP (P7)). During kinetic refolding t_1/2_ was the first time point at which half of the fluorescence was recovered. In each case, native samples were diluted into an equivalent volume of TNG buffer and read identically. The fraction folded, F_f_, is the fluorescence of the experimental divided by the native sample at each time point to correct for the minimal effects of dilution and photobleaching.

## Supporting Information

Figure S1 (1.77 MB TIF)
