# Supplementary figures and images for: Laboratory Evolution of Fast-Folding Green Fluorescent Protein Using Secretory Pathway Quality Control
Source: PLoS One. 2008 Jun 11;3(6):e2351. doi: 10.1371/journal.pone.0002351 (PMC2396501; doi:10.1371/journal.pone.0002351)

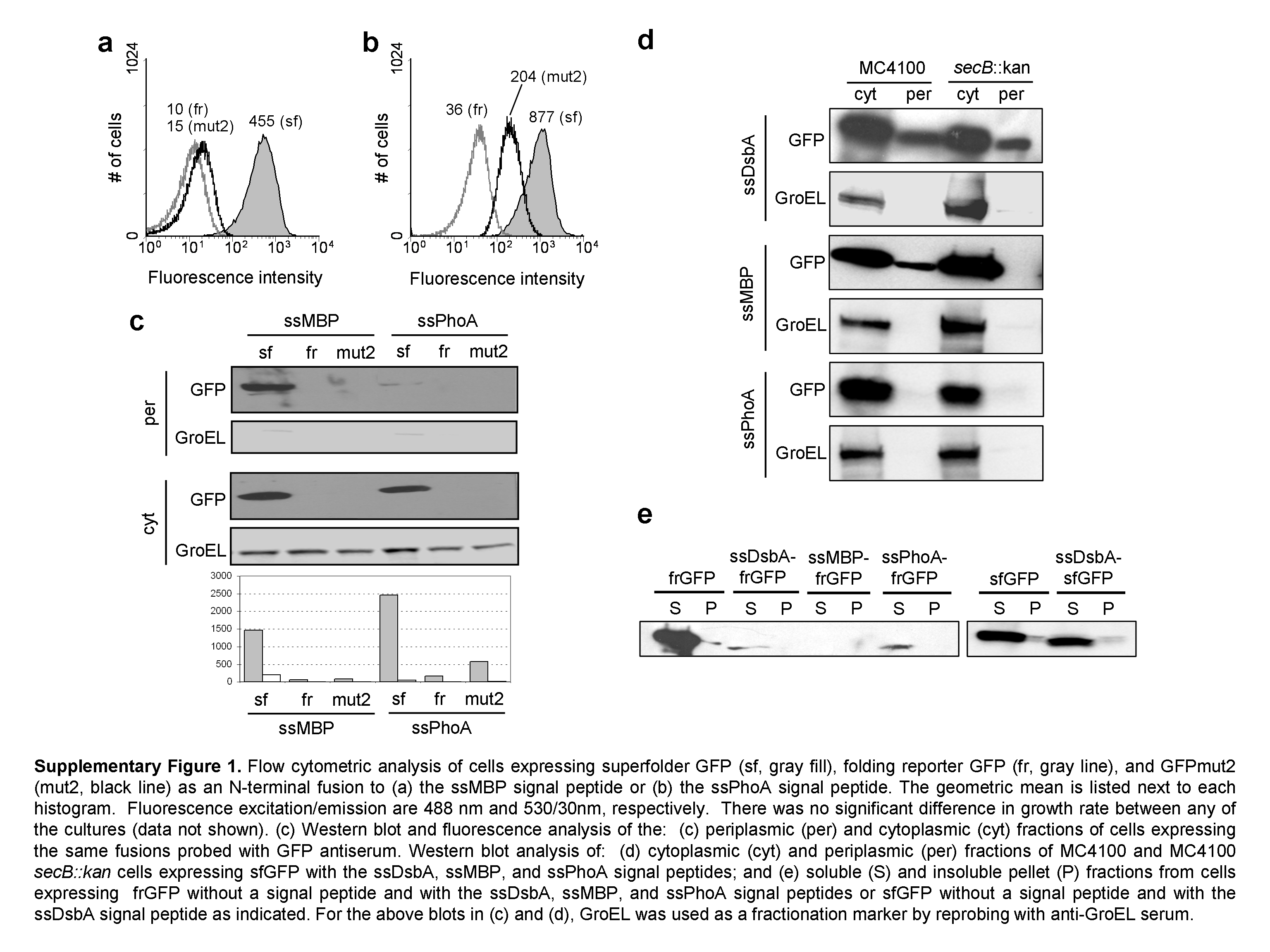

Supplement: Figure S1 — (1.77 MB TIF) [file pone.0002351.s001.tif]
